# A Novel Tightly Regulated Gene Expression System for the Human Intestinal Symbiont *Bacteroides thetaiotaomicron*

**DOI:** 10.3389/fmicb.2016.01080

**Published:** 2016-07-13

**Authors:** Nikki Horn, Ana L. Carvalho, Karin Overweg, Udo Wegmann, Simon R. Carding, Régis Stentz

**Affiliations:** ^1^Gut Health and Food Safety Programme, Carding's Lab, Institute of Food ResearchNorwich, UK; ^2^Department of Medicine, Norwich Medical School, University of East AngliaNorwich, UK

**Keywords:** expression system, inducible promoter, PUL, mannan, *Bacteroides thetaiotaomicron*

## Abstract

There is considerable interest in studying the function of *Bacteroides* species resident in the human gastrointestinal (GI)-tract and the contribution they make to host health. Reverse genetics and protein expression techniques, such as those developed for well-characterized *Escherichia coli* cannot be applied to *Bacteroides* species as they and other members of the Bacteriodetes phylum have unique promoter structures. The availability of useful *Bacteroides*-specific genetic tools is therefore limited. Here we describe the development of an effective mannan-controlled gene expression system for *Bacteroides thetaiotaomicron* containing the mannan-inducible promoter–region of an α-1,2-mannosidase gene (BT_3784), a ribosomal binding site designed to modulate expression, a multiple cloning site to facilitate the cloning of genes of interest, and a transcriptional terminator. Using the *Lactobacillus pepI* as a reporter gene, mannan induction resulted in an increase of reporter activity in a time- and concentration-dependent manner with a wide range of activity. The endogenous *BtcepA* cephalosporinase gene was used to demonstrate the suitability of this novel expression system, enabling the isolation of a His-tagged version of BtCepA. We have also shown with experiments performed in mice that the system can be induced *in vivo* in the presence of an exogenous source of mannan. By enabling the controlled expression of endogenous and exogenous genes in *B. thetaiotaomicron* this novel inducer-dependent expression system will aid in defining the physiological role of individual genes and the functional analyses of their products.

## Introduction

Inducible expression systems are essential molecular tools designed to perform phenotypic examinations of deletion mutants complemented for one or several genes of interest with the aim of defining the role and function of the expressed protein(s). This procedure often requires the use of finely tuned gene regulation which is particularly critical for the study of genes exhibiting toxic effects when expressed above normal physiological levels.

There is considerable interest in using dominant members of the human intestinal microbiota such as *B. thetaiotaomicron* as a model system to understand and identify the bacterial factors that are important for successful colonization of the GI-tract and the establishment of microbe-host mutualism. *B. thetaiotaomicron* has the capacity to utilize a wide variety of otherwise indigestible dietary plant polysaccharides and host-derived glycans as a source of carbon and energy (Salyers et al., 1977). The functional analysis of *Bacteroides* genes and metabolic pathways is however constrained by a lack of genetic tools. The genetic tools developed for model microorganisms such as *Escherichia coli* are of very limited use for *Bacteroides* species that have promoter structures with a unique consensus sequence (Bayley et al., 2000) recognized by its core RNA polymerase and its own unusual primary sigma factor (Vingadassalom et al., 2005). Parker and Smith circumvented this obstacle by engineering a *B. fragilis* promoter to which an *E. coli* promoter-regulatory region was added to construct an isopropyl β-D-1-thiogalactopyranoside (IPTG)-inducible expression system adapted to *B. fragilis* (Parker and Jeffrey Smith, 2012). However, the range of activity of this engineered *Bacteroides* expression vector is only 7–10-fold, which is a limiting factor when larger changes of protein expression are needed. Recently, Mimee et al. (2015) developed this system further by investigating the positional effects of operator sites on gene expression. This strategy produced IPTG-inducible promoters eliciting up to 22-fold changes in gene expression. The authors expanded further the range of gene expression (up to 10,000-fold range) using combinations of constitutive *Bacteroides*-derived promoters and ribosome binding sites.

Here we describe the development of an inducible gene expression system for *B. thetaiotaomicron* that is based upon an endogenous mannan-inducible promoter. This system has proven effective for the controlled expression of the β-lactamase BtCepA resulting in *B. thetaiotaomicron* displaying a broad range of minimum inhibitory concentrations (MIC) of ampicillin in a dose-dependent manner which correlated with mannan-induced BtCepA enzyme levels.

## Materials and methods

### Bacterial strains, growth conditions, and media

All *E. coli* and *B. thetaiotaomicron* strains used in this study are listed in Table 1*. Bacteroides* strains were grown in either Brain Heart Infusion (BHI) medium (Oxoid/Thermo Fisher, Basingstoke, UK) supplemented with 0.001% hemin (BHIH) or in *Bacteroides* Adapted Defined Medium (BDMA, See Table S1) adapted from (Martens et al., 2008). Antibiotics were added as selective agents when appropriate: gentamicin (200 μg/ml), erythromycin and tetracycline (5 μg/ml). Cultures were incubated under anaerobic conditions at 37°C. *E. coli* strain JM109 was used for routine cloning and DNA manipulations. Cultures were grown in Luria Bertani (LB) medium at 37°C. Ampicillin (200 μg/ml) was added when appropriate. The *E. coli* strain J53/R751 was supplemented with 200 mg/ml trimethoprim when grown for 18 h. Plasmids constructed in *E. coli* were mobilized into *Bacteroides* strains by triparental mating using J53/R751 as the conjugal helper strain (Shoemaker et al., 1986). Electrocompetent *E. coli* cells were prepared and transformed by the method of Sambrook and Russell (2001).

**Table 1:** **List of bacterial strains and plasmids used in this study**.

**Species**	**Strain**	**Plasmid**	**Promoter**	**RBS**	**Gene overexpressed**	**Antibiotic selection**	**References**
*E. coli* JM109	GH019	pGH001	P1	Low	*pepI*	Amp	Wegmann et al., 2013
	GH079	pGH020	P1	Med		Amp	Wegmann et al., 2013
	GH081	pGH022	P1	Med	*pepI*	Amp	Wegmann et al., 2013
	GH188	pGH063		Med	*pepI*	Amp/Tet	This study
	GH303	pGH117	P3784	Med		Amp/Ery	This study
	GH317	pGH122	P3784	Low		Amp/Ery	This study
	GH349	pGH141	P3784	Low		Amp/Ery	This study
	GH350	pGH142	P3784	Med		Amp/Ery	This study
	GH405	pGH125med	P3784	Med		Amp/Ery	This study
*B. thetaiotaomicron*	VPI-5482						DMSZ Collection
	GH221^a^					Tet	Stentz et al., 2015
	GH189	pGH022	P1	Med	*pepI*	Tet	Wegmann et al., 2013
	GH190	pGH063		Med	*pepI*	Tet	This study
	GH193	pGH066	P3784	Med	*pepI*	Tet	This study
	GH287	pGH105	P3784	Low	*pepI*	Tet	This study
	GH288	pGH106	P3784	Med		Tet	This study
	GH289	pGH107	P3784	Low		Tet	This study
	GH359	pGH117	P3784	Med		Ery	This study
	GH360	pGH122	P3784	Low		Ery	This study
	GH361^a^	pGH141	P3784	Low	*BtcepA*	Ery	This study
	GH364^a^	pGH142	P3784	Med	*BtcepA*	Ery	This study
	GH402^a^	pGH150	P3784	Med	*BtcepA*[His]_6_	Ery	This study

a B. thetaiotaomicron ΔBtcepA strain.

Amp, ampicillin; Tet, tetracycline; Ery, erythromycin.

### Identification of inducible promoters

The Gene Expression Omnibus data repository (NCBI; http://www.ncbi.nlm.nih.gov/geo/) was consulted in order to identify genes exhibiting low basal expression in the presence of glucose but were highly induced in the presence of another carbon-source in *B. thetaiotaomicron* VPI-5482. The Series GSE11962 [growth of *B. thetaiotaomicron* on purified host mucosal glycans and glycan fragments (Martens et al., 2008)] was used for the identification of promoters.

### Plasmid constructions

#### Removal of the P1 promoter from pGH022

First, a 228-bp fragment was amplified from pGH022 (See Table 1 for a list of plasmids) using primer f-noPpepI (See Table S2 for a list of primers), which contains the restriction sites *Sph*I, *Nco*I, *Xho*I, and *Eco*47III and incorporates the transcription initiation site (TIS) and a ribosome binding site for medium level protein expression (RBS_med_; original name RBS_xyl−20_ in Wegmann et al., 2013), and primer r-ppepI/*Not*I, located 157 bp downstream of the start codon of *pepI*. This fragment was digested with *Sph*I and *Not*I, and cloned into *Sph*I- and *Not*I-digested pGH022 to create pGH063.

#### Insertion of the BT-3784 promoter (P_3784_)

The 272-bp region located upstream of gene BT_3784 (putative alpha-1,2-mannosidase) was amplified from *B. thetaiotaomicron* VPI-5482 genomic DNA using primers f-3784_3786 and r-3784_3786_sp. This fragment was digested with *Sph*I and cloned into *Sph*I- and *Eco*47III-digested pGH063 to create pGH066, thereby inserting the BT-3784 promoter.

#### Replacing the P1-RBS_med_ region of pGH022 with P_3784_-RBS_low_

To replace the region spanning the P1 promoter to the RBS_med_ sequence of pGH022 with the P_3784_ promoter and a low level protein expression ribosome binding site equivalent [RBS_low_; original name Shine Delgarno 8 (SD8) in (Wegmann et al., 2013)], splice overlap extension PCR was employed. To this end, Amplicon 1 was generated from pGH066 using primers f-RBS_low_-pepI and primer r-3784_3786_sp. Amplicon 2 was generated from pGH001 using primers r-3784_RBS_low_ and r-ppepI/*Not*I. Using a mixture of amplicon 1 and 2 as a template and primers r-3784_3786_sp and r-ppepI/*Not*I, splice PCR was performed. A 452-bp *Sph*I- and *Not*I-digested fragment of this PCR product was then used to replace the corresponding 329-bp fragment of pGH022 to create pGH105.

#### Expression vectors pGH106 and pGH107

To construct the expression vectors pGH106 (medium) and pGH107 (low) insert sequences were amplified from pGH066 using either primer f-RBS_med__MCS or primer f-RBS_low__MCS, together with primer r-3784_3786_sp. A 297-bp (RBS_med_) or 292-bp (RBS_low_) *Sph*I- and *Nco*I- digested fragment of each PCR product was used to replace the corresponding 169-bp fragment of pGH020.

#### Replacing tetQ with ermF

**Figure 1:**
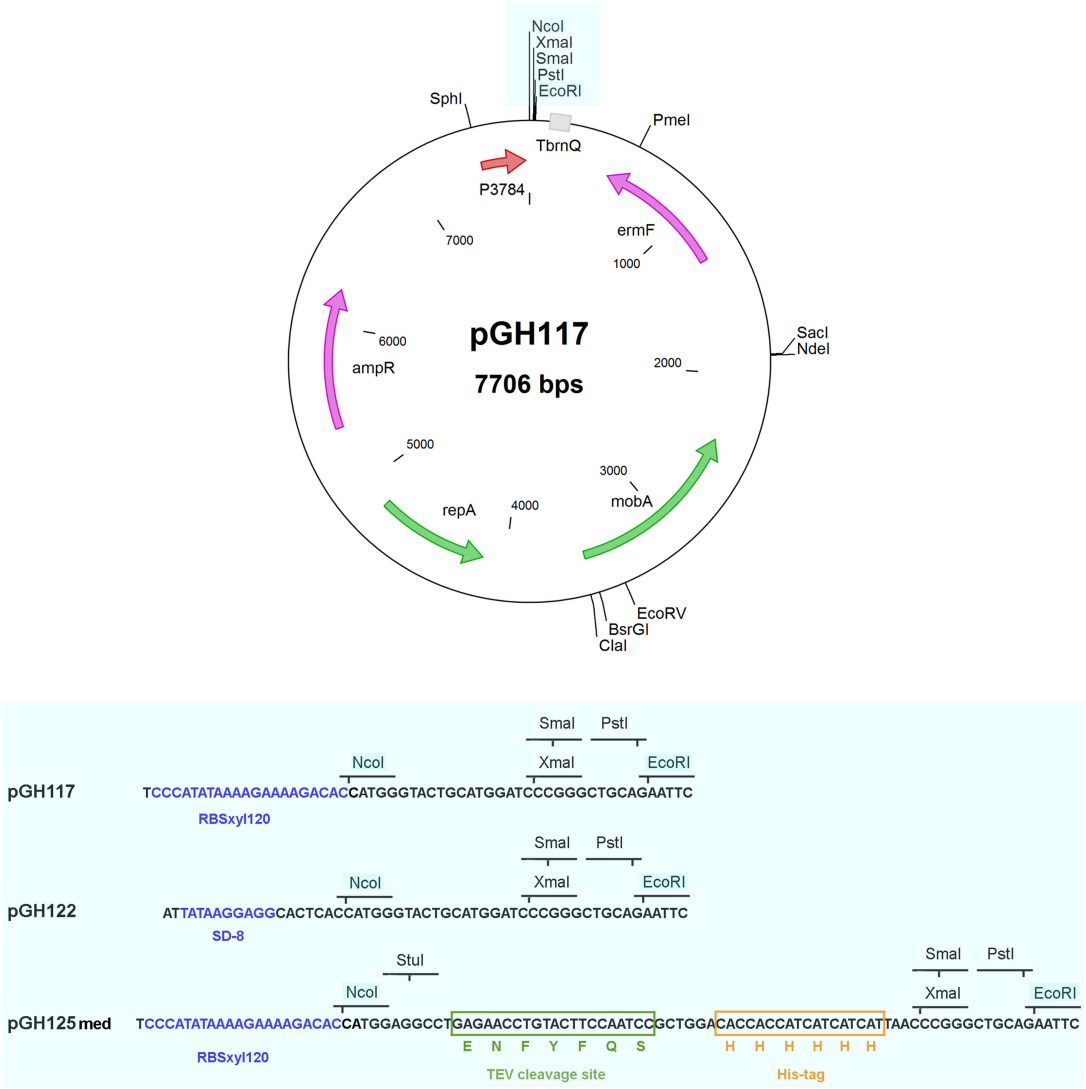
**Plasmid map of the inducible expression vectors pGH117, pGH122, and pGH125med**. The sequence of the multiple cloning site flanked by the *Nco*I and *Eco*RI restriction sites is shown below for each vector. RBSxyl120 is a ribosome binding site providing medium level protein expression and SD-8 allows low level expression.

The primer pair ccr_amont2 and ccr_aval2 was used to amplify a 1500-bp region carrying ermF from the plasmid pFD516 (Smith et al., 1995). The ermF fragment was digested with NdeI and cloned into NdeI-digested (blunted) and NsiI-digested pGH106 or pGH107 to replace the existing 1839-bp fragment of each, to create pGH117 (RBS_med_) and pGH122 (RBS_low_), respectively, (Figure 1).

#### Addition of the Tev/6xHis-tag linker to pGH117

Primers Tev-His6_linker_5′ and Tev-His6_linker_3′ were annealed to form a 66-bp *Nco*I-*Stu*I-*Sma*I linker with *Nco*I and *Sma*I compatible ends enabling the direct cloning of this fragment into *Nco*I- and *Sma*I-digested pGH117, to create pGH125med (Figure 1). The presence of this linker supplies the option to clone a gene of interest into the *Nco*I and *Stu*I sites of pGH125med, the result of which is the addition of an epitope recognition site for the TEV protease enzyme followed by a 6xHis-tag to the C-terminus of the final expressed protein.

### Cloning of native and recombinant BtCepA

The primer pair Lactamase_F and Lactamase_*Eco*RI_R was used to amplify an 885-bp region from *B. thetaiotaomicron* VPI-5482 genomic DNA encoding *BtcepA*. The *BtcepA* fragment was digested with *Eco*RI before cloning it into the *Nco*I-digested (blunted) and *Eco*RI-digested pGH122 (RBS_low_) or pGH117 (RBS_med_) to create pGH141 and pGH142, respectively. In the case of recombinant *BtcepA* the primer pair Lactamase_F and B-Lact_nostop_R was used to amplify an 875-bp region encoding *BtcepA*[His]_6_ from *B. thetaiotaomicron* VPI-5482 genomic DNA. The *BtcepA*[His]_6_ fragment was cloned into the *Nco*I-digested (blunted) and StuI-digested pGH125med (RBS_med_[His]_6_) to create pGH150.

### Enzyme assays and protein determination

The *pepI* gene of *Lactobacillus delbrueckii* subsp. *lactis* encoding peptidase I and the *BtcepA* gene encoding a *B. thetaiotaomicron* β-lactamase were used as reporters to study promoter activity in *B. thetaiotaomicron* in response to the mannan inducing agent. The *B. thetaiotaomicron* strains under study were grown in BDMA. For the PepI assay, cultures (200 ml) were grown in duplicate to an optical density at 600 nm of 0.5 before induction. To determine the optimal inducer concentration, mannan was added to 20 ml aliquots at a final concentration of 0, 0.1, 1.0, 5.0, 10, 50, or 100 mg/L. Following a 2-h induction period cells were collected by centrifugation at 6000 g for 15 min at 4°C. The preparation of *B. thetaiotaomicron* cell-free extracts was performed as previously described (Wegmann et al., 2013). To determine the optimal induction time, mannan was added to a final concentration of 100 mg/l and incubation continued. Subsequently, 20 ml samples were collected over time and the cells harvested as described above. The protein concentration and peptidase I activity was determined according to the method described by Wegmann et al. (2013). Specific activity is expressed as nanomoles of p-nitrophenol released from the chromogenic substrate per milligram protein per minute. For the β-lactamase assay, cultures (200 ml) were grown in duplicate to an optical density at 600 nm of 0.5 before inducing 20 ml aliquots with varying levels of mannan (See Table 2) or 5 mg/L of cefotaxime. Periplasmic proteins were prepared as described by Stentz et al. (2015). β-lactamase activity was assessed spectrophotometrically by hydrolysis of nitrocefin according to the manufacturer's instructions (Calbiochem). The means and standard deviations presented are based upon two biological replicates with three technical replicates each.

**Table 2:** **BtCepA β-lactamase activity after mannan induction**.

**Strain^a^**	**Description**	**Mannan (mg/l)**	**nmol/mg/min**	**Fold-Induction^c^**
GH359	WT (P_3784__low)	0	304.5 ± 41.3	–
		0 [Cef_5_]^b^	642.9 ± 70.9	–
GH360	Δ*BtcepA* (P_3784__low)	50	3.4 ± 2.0	–
GH361	Δ*BtcepA* (P_3784__low::*BtcepA*)	0	11.4 ± 0.3	1.0
		50	131.7 ± 4.6	11.6
		100	177.1 ± 26.9	15.6
		250	191.1 ± 25.5	16.8
GH364	Δ*BtcepA* (P_3784__med::*BtcepA*)	0	1016.9 ± 13.4	89.4
		5	5872.3 ± 350.4	516.4
		50	13133.2 ± 503.7	1154.9

a The strains are also described in Table 1.

b Addition of 5 mg/l cefotaxime.

c Induction factor = the mean value obtained in the indicated conditions/the mean value obtained for the non-induced GH361 strain (11.4).

### BtCepA purification

For the purification of BtCepA[His]_6_ from *B. thetaiotaomicron*, cultures (100 ml) were grown to an optical density at 600 nm of 0.5 before inducing for 2-h with 100 mg/ml of mannan. Cells were collected in two aliquots of 50 ml by centrifugation at 3500 g for 10 min at ambient temperature. Each 50 ml cell pellet was processed to extract its periplasmic proteins as described by Stentz et al. (2015) with the exception that a final volume of 0.4 ml of ice-cold MgSO_4_ 5 mM solution was used. To the recovered osmotic shock fluid, 63 ml of 10x concentrated lysis buffer (NaH_2_PO_4_ 0.5 M, NaCl 0.3 M, imidazole 0.1 M, pH8) was added before the final volume was adjusted to 600 ml using 5 mM MgSO_4_. Contaminating cell debris was removed by centrifuging at 12,000 g for 15 min at 4°C before continuing the purification process using a Ni-NTA spin Kit (Qiagen, UK). Purification of BtCepA[His]_6_ was performed under native conditions according to the manufacturer's instructions. Selected spots were picked on the single band obtained after SDS-PAGE and trypsin-digested using the ProPick Spot Picker (Genomic Solutions) and ProGest protein digestion robot (Genomic Solutions) prior to peptide mass fingerprinting on an Ultraflex II MALDI-TOF-TOF (Bruker) using an offline version of Mascot (Matrix Sciences) searching against *B. thetaiotaomicron* sequences.

### Animal experiments

*B. thetaiotaomicron* was established within the intestine of 7-week-old male C57BL/6J WT mice housed in a conventional animal facility after treating for 3 days with 1 mg/ml ampicillin and 1 mg/ml neomycin administered via drinking water. Bacterial suspensions of the GH361 strain [1 × 10^8^ colony-forming units (CFU) in 100 μl of phosphate buffered saline] were administrated by orogastric gavage to a total of eight mice four of which were given access to drinking water containing 2.5% (v/v) mannan-oligosaccharide supplement (ActiveMOS, Orffa, Werkendam, Netherlands) prepared treating the powder (10% w/v) with 1% NaOH at 100°C for 1 h, cooling and neutralizing to pH7 with dilute HCl solution (Huang et al., 2010). We note that addition of the resulting mannan solution to growth cultures at a concentration of 2.5% (v/v) fully promoted the mannan-induced system. After 3 days all mice were injected i.p. with ceftriaxone (0.4 mg/mice), which was repeated every 2–3 days. Fresh feces were collected into sterile containers, weighed and homogenized in PBS. Serial dilutions of the supernatants were plated onto BHI supplemented with 1.5 μg/ml tetracycline and 5 μg/ml erythromycin and incubated anaerobically at 37°C with CFU counted 48 h later. The study was reviewed and approved by the Animal Welfare and Ethical Review Body (AWERB, University of East Anglia, Norwich, UK) and was conducted within the provisions of the Animals (Scientific Procedures) act 1986. To confirm that the chow (Rat and Mouse n°3 breeding, Special Diet Services) used in this experiment did not contain α-mannan or derivatives able to stimulating the system, the chow was subjected to a mannan extraction process performed in alkaline conditions, as previously described (Huang et al., 2010). The resulting solution was added at a 1:5 dilution to fresh medium with no stimulation of the mannan-inducible P_3784_ promoter observed after growth of the cells.

## Results and discussion

### Designation of a *B. thetaiotaomicron* inducible promoter

To develop an inducible gene expression system that can be used in *B. thetaiotaomicron*, publicly available microarray data were examined for inducible genes exhibiting a low basal expression level, and a high expression level (increased by more than 100-fold) in the presence of the inducer. Genes repressed by glucose and induced in the presence of other defined carbon-sources were identified using the NCBI Gene Expression Omnibus web tool (http://www.ncbi.nlm.nih.gov/geo/, series GSE11962) and obtained by transcriptional profiling of *B. thetaiotaomicron* with the aim of establishing the mechanisms underlying host glycan foraging (Martens et al., 2008). Among possible candidates the BT_3784 gene encoding an α-1,2-mannosidase (Xu et al., 2003), that is part of the polysaccharide utilization locus (PUL) 68 (4) later named MAN-PUL2 (Cuskin et al., 2015) and is induced by the yeast polysaccharide α-mannan, was selected. A 272-base pair DNA fragment located upstream of the BT_3784 gene (Presentation 1) was cloned in front of the peptidase I reporter gene (Klein et al., 1994) in an expression vector created to express medium levels of protein in *Bacteroides* species (Wegmann et al., 2013). The resulting construct hosted by *B. thetaiotaomicron* created the strain GH193, which was tested for its capacity to conditionally express PepI used as a reporter.

### Effect of carbon source on BT_3784 promoter activity

**Figure 2:**
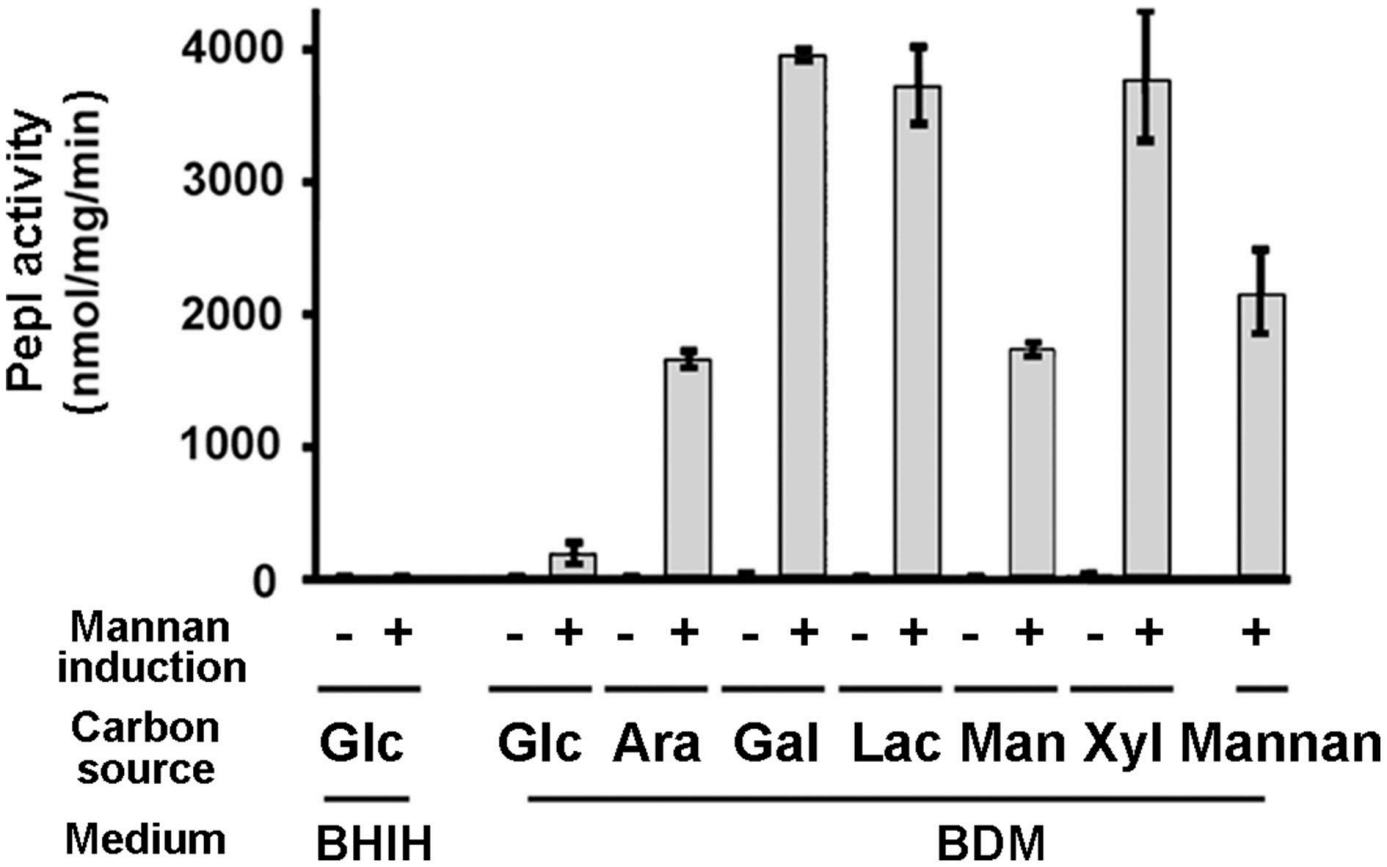
**Expression of *pepI* under the control of P_3784_ in the presence of different carbon sources**. The *B. thetaiotaomicron* strain GH193 containing the *pepI* gene under the dependence of the P_3784_ promoter was grown in the BHI complex medium to which hemin was added (BHIH). BHI is commercially supplied and contains 0.2% glucose. GH193 was also grown in minimal medium (BDMA) on different carbon sources (0.5%) to which 50 mg/L mannan (extracted from *S. cerevisiae*) was added where indicated.

The reporter activity of GH193 was tested after growth in rich medium (BHIH) and in minimal medium (BDMA) supplemented with different carbon-sources, in the presence or absence of the exogenous mannan (Figure 2). In minimal media the growth rate of strain GH193 was similar in all cases independent of the carbon source present with the exception of mannan for which GH193 exhibited a slower growth with a generation time of 130 min compared to 90 min under the other conditions. In rich media, reporter activity in GH193 did not significantly increase upon mannan induction. As BHI (to which hemin (H) was added) contains 0.2% glucose, this suggests that glucose represses the expression of BT_3784 as reported by Martens et al. (2008), despite the presence of mannan. However, in minimal medium supplemented with glucose as the major carbon source mannan induction resulted in an increase of PepI activity of about 15-fold (Figure 2) indicating that additional sources of repression are most likely present in BHI mixture.

Mannan-induced PepI activity for cells grown on galactose, lactose and xylose was increased 110 to 120-fold compared to the respective control cultures lacking mannan (Figure 2). The PepI activities measured for cells grown on arabinose and mannose in the presence of mannan, although lower, were still 87 and 109 times higher, respectively, than control cultures lacking mannan (Figure 2). Not surprisingly, despite a slower growth rate, cells grown on mannan exhibited PepI activity comparable to the activities obtained for mannan-induced cells grown on arabinose and mannose.

Among the carbon sources tested in this study, only glucose negatively affected mannan induction of the BT_3784 promoter (P_3784_). Glucose negatively regulates expression of α 1,2 mannosidase as previously observed in other *Bacteroides* species for the expression of other glycosidases such as β-glucosidase in *B. ruminicola* (Strobel and Russell, 1987) or xylanase in *B. ovatus* (Hamady et al., 2008).

Of note, low levels of PepI activity were detectable in the absence of mannan consistent with previous data showing low level of *B. thetaiotaomicron* PUL expression in conditions lacking the relevant substrates (Sonnenburg et al., 2006; Martens et al., 2009; Sonnenburg et al., 2010). Of significance, the monosaccharide mannose, one of the major end-products of mannan enzymatic hydrolysis (Düsterhöft et al., 1993), does not affect expression of the α 1,2 mannosidase gene although this substrate has been shown to have an inhibitory effect on the utilization of several hexoses including glucose and pentoses in *B. thetaiotaomicron* (Degnan and Macfarlane, 1995).

### Effect of mannan concentration and induction time on promoter activity

**Figure 3:**
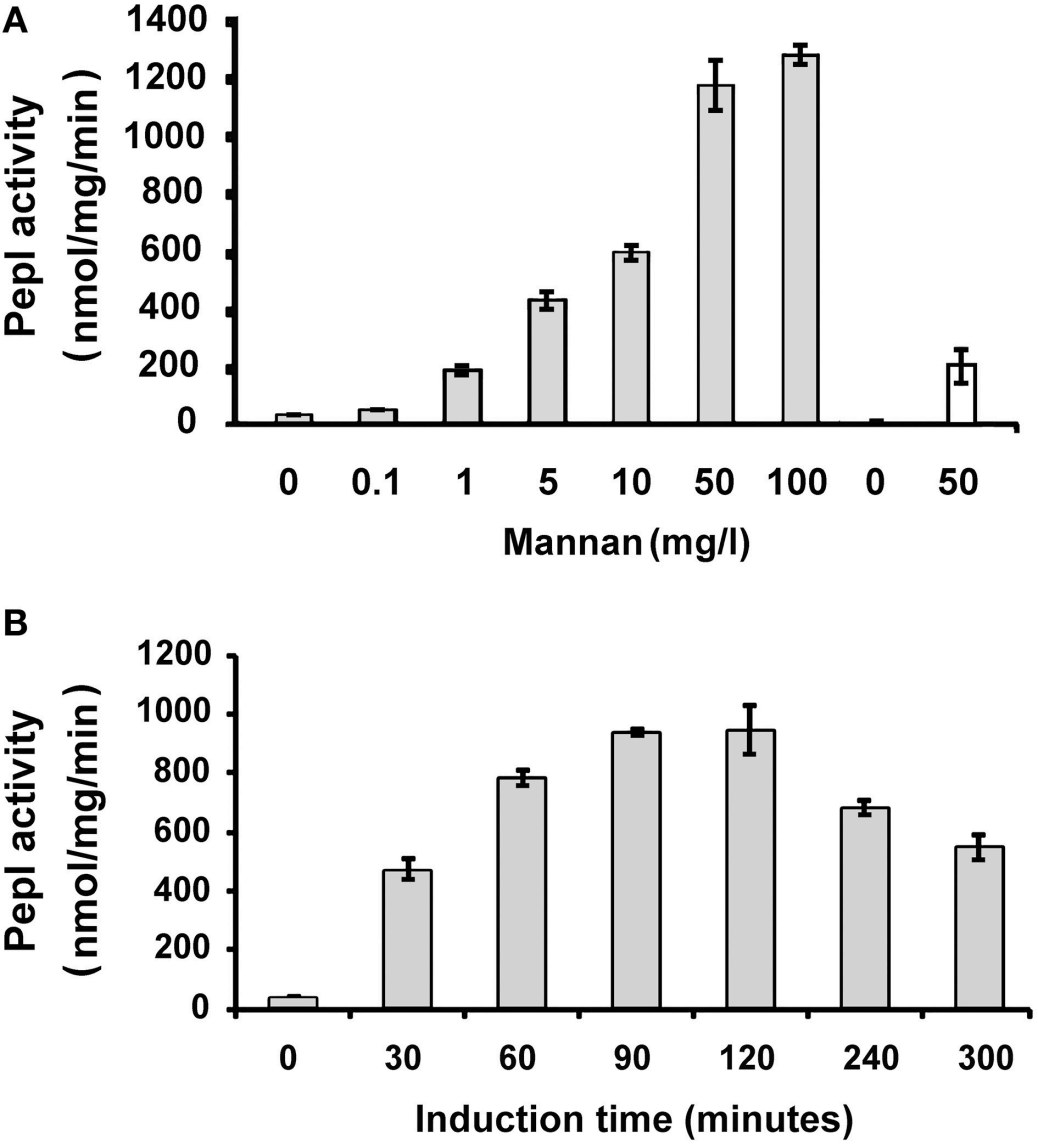
**Determination of optimal conditions for mannan-induced gene expression. (A)**. The *B. thetaiotaomicron* strain GH193 (light gray) which expresses medium-levels of PepI under the control of the P_3784_ promoter was incubated in the presence of increasing concentrations of mannan and the specific activity of PepI was determined. The GH287 strain (white) contained a vector designed for low expression levels of PepI. **(B)**. GH193 was grown in the absence and presence of mannan (100 mg/l) and PepI activity was measured in protein extracts from samples collected at different time points post induction.

The response of P_3784_ to increasing concentrations of mannan was tested for cells grown in minimal medium containing xylose. The strains tested were GH193, containing a *Bacteroides* expression vector that contains a translation initiation signal designed for medium expression (Wegmann et al., 2013) and GH287, containing a vector designed for low expression (Wegmann et al., 2013) to ensure that the widest range of expression levels were covered with the lowest basal activity. The basal level activity of P_3784_ measured for GH287 was five-fold lower than for strain GH193 (Figure 3A). For both strains GH193 and GH287, PepI activity reached its peak at mannan concentrations as low as 50 mg/l (Figure 3A).

To further optimize the conditions of induction, the minimum induction time required to generate the highest level of reporter activity was determined. The response of the promoter was assessed over a time-course of mannan-induction in strain GH193 incubated with 50 mg/L mannan. PepI activity was detectable after 30 min and continued to increase thereafter with the maximal activity reached after 90 min (Figure 3B). In the case of GH287, the strain containing the low-RBS-P_3784_ construct, maximal activity was also reached after 90 min (data not shown).

To facilitate the controlled expression of genes of interest in *Bacteroides*, plasmids were constructed that contain a multiple cloning site in the position of the *pepI* reporter resulting in plasmid pGH106 for medium expression and pGH107 for low expression.

### Mannan-controlled resistance to ampicillin and BtCepA purification

Since optimization of this novel gene expression system was based upon the use of a heterologous reporter gene, we chose to validate the system by determining if it can be used to study *B. thetaiotaomicron* genes. For this purpose, we undertook the complementation of a deletion mutant of *BtcepA* (Stentz et al., 2015), a cephalosporinase gene involved in the resistance of bacterial cells to β-lactam antibiotics. The β-lactamase activity was measured after 2 h of induction with increasing concentrations of mannan (Table 2). The activities in the non-induced and cefotaxime-induced (Stentz et al., 2015) wild-type strain were also measured and used as references. Use of increasing concentrations of mannan with the low-expression system gradually restored up to two-thirds of β-lactamase activity produced in the wild-type strain. With the medium expression system graduated and increasing levels of β-lactamase activity were produced which eventually exceeded the native expression levels of *BtCepA*. The use of the low and medium variants permitted the induction of CepA activity values ranging from 1 to 1155 fold activity values. The basal level measured for the low expression system was negligible since it represented only 2.7% of the activity measured for the wild-type control strain.

To establish how these measured activities translate into resistance to β-lactam antibiotics the MIC of the β-lactam antibiotic ampicillin was determined for each variant strain grown in the presence or absence of mannan. The measured MIC values closely correlated with β-lactam activities measured in the corresponding strains (Table 3) with values ranging from 4 to 2048 mg/L.

**Table 3:** **Mannan-induced BtCepA expression increases the resistance of *B. thetaiotaomicron* to ampicillin**.

**Strain**	**Mannan^a^**	**Ampicillin (mg/l)**^b^												
		0	1	2	4	8	16	32	64	128	256	512	1024	2048
														
WT	+													
														
Δ*BtcepA*	+													
														
Δ*BtcepA* (P_3784__low)	−													
														
Δ*BtcepA* (P_3784__low)	+													
														
Δ*BtcepA* (P_3784__med)	−													
														
Δ*BtcepA* (P_3784__med)	+													
														

a Addition of 100 mg/l mannan to bacterial cultures with an OD_600_ of 0.5 and the cells were incubated for 16 h.

b Determination of ampicillin minimum inhibitory concentration (MIC) for the different B. thetaiotaomicron strains. The horizontal bars in black cover the ampicillin concentrations that permit growth for each of the strains. The results are representative of two independent experiments.

**Figure 4:**
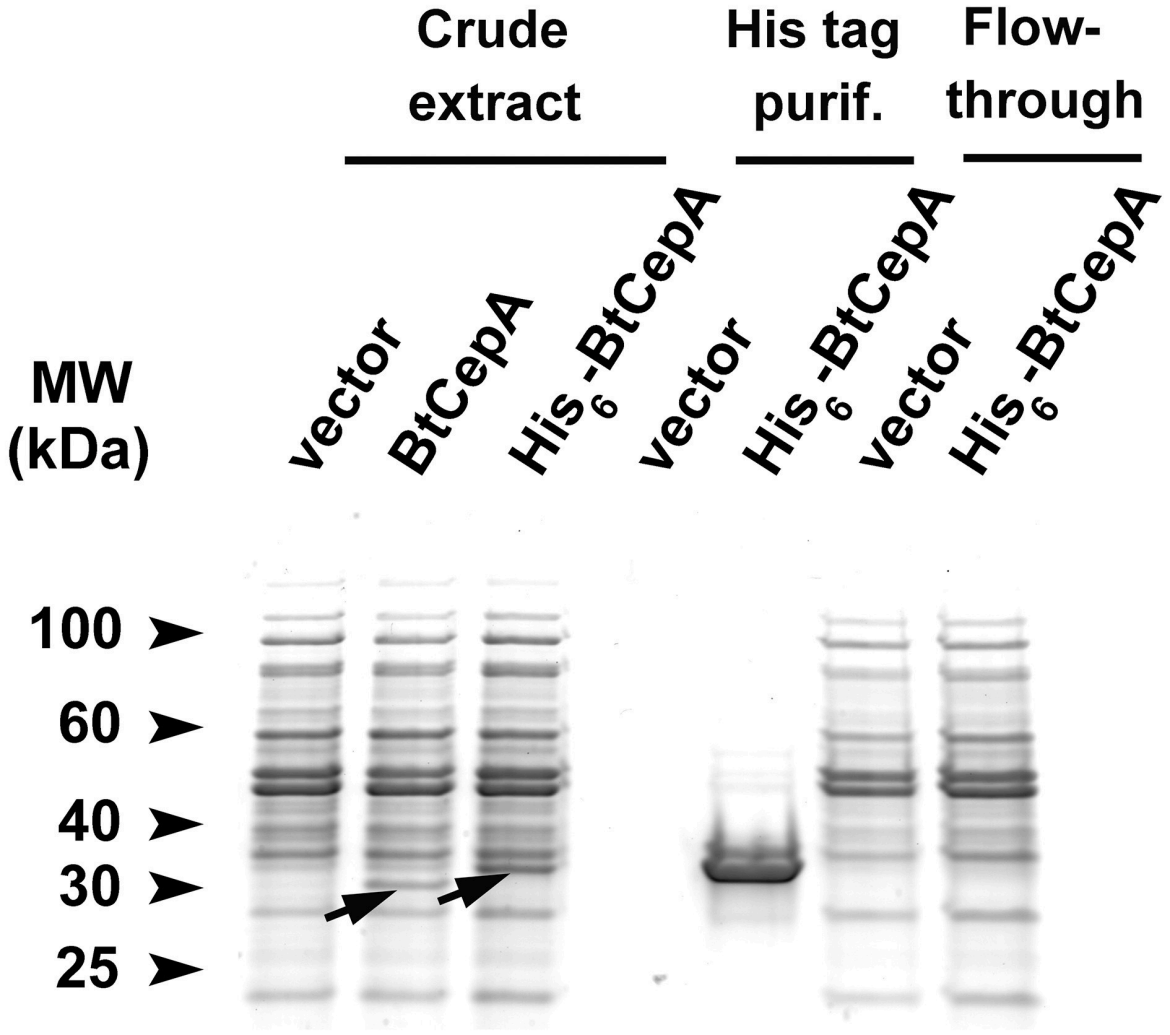
**SDS-PAGE of periplasmic protein extracts before and after Ni-NTA affinity chromatography**. MW, protein ladder; vector, extract from GH359 strain which contains the empty vector; BtCepA, extract from GH364 which expresses the native BtCepA; BtCepA-His_6_, extract from GH402 which expresses the C-terminally His-tagged BtCepA. Flow-through was collected during the binding step of purification. The arrows indicate the bands corresponding to the native BtCepA and the higher molecular weight BtCepA [His]_6_.

We also utilized our system for the expression of a recombinant His-tagged BtCepA protein. To this end, the C-terminus of the *BtcepA* coding region was fused to the hexahistidine affinity tag encoded on pGH125med. The strain GH402 containing the construct was induced for 5 h in the presence of mannan and the cell periplasmic fraction was obtained. Recombinant BtCepA containing a C-terminal His-tag was purified by affinity chromatography and loaded on a SDS-PAGE (Figure 4). As a control, native BtCepA was expressed under the same conditions with a SDS-PAGE-resolved band corresponding to the protein detected in periplasmic extracts (Figure 4). A band of a slightly higher molecular weight was detected for the BtCepA-His_6_ fusion protein attributable to the protein tag. After purifying and concentrating the eluted band, a single species corresponding to BtCepA-His_6_ was obtained whereas no comparable band was detected for the empty vector control with no protein corresponding to BtCepA-His_6_ detected in the flow-through (Figure 4). Using peptide mass fingerprinting, the band was identified as BtCepA.

Thus, we have developed an efficient system that allows phenotypic restoration of *B. thetaiotaomicron* deletion mutants in a dose-dependent manner as well as purification of recombinant proteins produced in their native host, a context within which recombinant proteins are more likely to retain their native characteristics.

### Mannan-induced BtCepA expression in the mouse gut

“α-mannan is a fungal cell wall glycan that contains α -mannosidic linkages similar to those found in the core regions of N-linked glycans present on secreted mucus and epithelial surfaces” (Martens et al., 2011). However, expression levels of the PUL genes involved in mannan utilization in mice fed a diet lacking exogenous α-mannan were only partial when compared to other PULs showing high activity *in vivo* (Martens et al., 2008). We therefore decided to test the effectiveness of the mannan-induced system *in vivo*.

**Figure 5:**
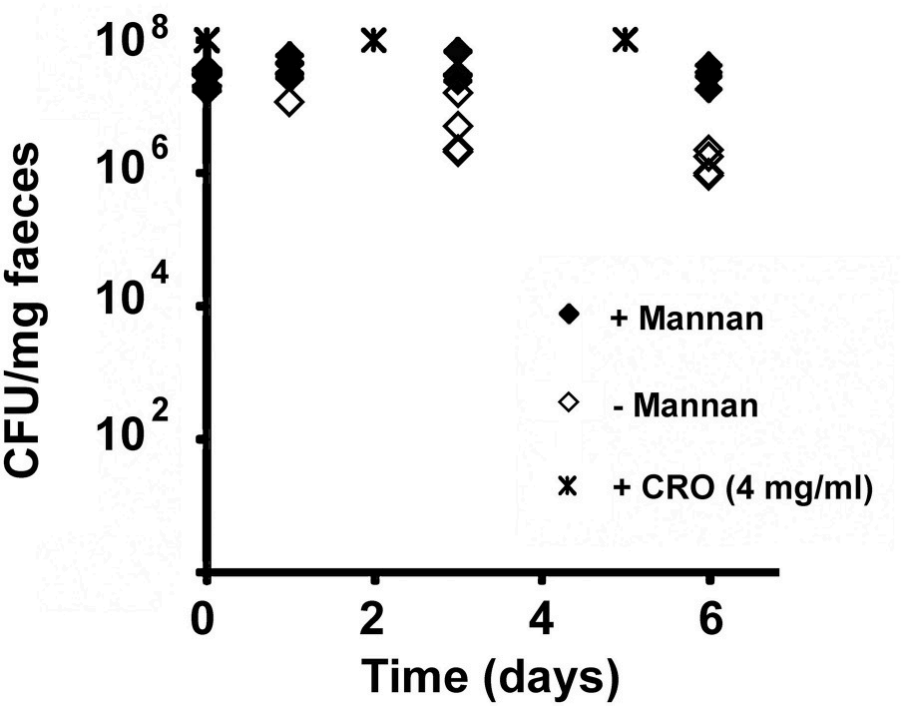
***In vivo* induction of ceftriaxone resistance using exogenous α-mannan**. Crosses indicate times of i.p. injections. GA: Group A of mice received mannan in their drinking water. GB: Group B of mice which were given access to regular drinking water. CRO: ceftriaxome.

To achieve this, the strain GH361 containing the *BtCepA* gene controlled by the inducible low-expression system (Table 1) was administered by oral gavage to two groups of four mice. Mannan was given to one of the groups (group A) via the drinking water and after 2 days both groups of mice were similarly colonized with GH361 as seen by counting the CFU in fecal samples (Figure 5) indicating that exposure to mannan did not impact on the ability of *B. thetaiotaomicron* to colonize the mouse intestine. Both groups of mice were challenged every other day with ceftriaxone (0.4 mg), a third-generation cephalosporin that is excreted via the biliary route and affects the colonic microbiota (Arvidsson et al., 1982). The MICs of ceftriaxone determined for GH361 were 8 mg/l when cells were grown in the absence of mannan and 64 mg/l when mannan was added to the growth medium.

Three successive doses of ceftriaxone had no effect on the levels of GH361 in group A that had been given mannan; however, the levels of GH361 that had not been given mannan (group B) decreased approximately 15-fold (Figure 5). Increasing further the amount of ceftriaxone to 0.8 mg showed that both groups were equally affected and population levels decreased significantly (data not shown). The moderate impact of mannan observed on GH361 resistance to ceftriaxone is most likely due to the induction of promoter activity by endogenous mannan and/or derivatives. The differences obtained in GH361 colonization between the groups with and without added mannan (*p* ≤ 0.01) suggests this system could be of use in *in vivo* studies. For example, it is conceivable to consider replacing the mannan promoter with PUL promoters regulated by plant-derived inducing agents such as arabinogalactan whose effectiveness was recently demonstrated (Mimee et al., 2015).

## Conclusion

A novel mannan-inducible gene expression system for use in *B. thetaiotaomicron* has been generated. We have shown that the gene BT_3784 is regulated in response to mannan and have combined the region located upstream of gene BT_3784 with two distinct ribosomal binding sites that allow the amount of gene expression to be tightly regulated. This mannan-controlled gene expression system is tunable via altering of the mannan concentrations and, in combination with ribosomal binding sites RBS_med_ and RBS_low_, generates more than a 1000-fold range of promoter activity. The system is remarkably efficient as it only requires concentration of mannan as low as 50 mg/l to be fully induced. The vectors described in this study constitute together a valuable tool for expressing *Bacteroides* genes and purifying their products, as well as studying *Bacteroides* cell physiology.

## Author contributions

Conceived and designed the experiments: RS, SRC. Performed the experiments: NH, KO, ALC. Analyzed the data: NH, ALC, KO, RS. Contributed reagents/materials/analysis tools: UW, NH. Obtained funding to support the work: SRC. Contributed to the writing of the manuscript: RS, SRC, KO, NH, ALC.

## Funding

This work was supported by institutional grants from Biotechnology and Biological Sciences Research Council (BBSRC; BB/J004529/1, SRC and BB/L004291/1, SRC).

### Conflict of interest statement

The authors declare that the research was conducted in the absence of any commercial or financial relationships that could be construed as a potential conflict of interest.
