# Supplementary material for: A Novel Tightly Regulated Gene Expression System for the Human Intestinal Symbiont Bacteroides thetaiotaomicron
Source: Front Microbiol. 2016 Jul 13;7:1080. doi: 10.3389/fmicb.2016.01080 (PMC4942465; doi:10.3389/fmicb.2016.01080)
Supplement: Supplementary file 1 [file Table1.DOCX]

Supplementary Material

**A Novel Tightly Regulated Gene Expression System for the Human Intestinal Symbiont** *Bacteroides* *thetaiotaomicron*

**Nikki Horn, Ana Lucia Carvalho, Karin Overweg, Udo Wegmann, Simon R. Carding, Régis Stentz***

*** Correspondence:** Corresponding Author: regis.stentz@ifr.ac.uk

| **Table S1.** BDM adapted (BDMA) composition. | | | | |  |  |  |  |  |  |
| --- | --- | --- | --- | --- | --- | --- | --- | --- | --- | --- |
|  | **Base medium** | | | **Final concentration** | | | | |  |  |
|  | Potassium phosphate (pH7.4) | | | | | | | 100 mM |  |  |
|  | (NH4)2SO4 | | | | | | | 8.5 mM |  |  |
|  | Hemin | | | | | | | 0.01 % |  |  |
|  | Histidine | | | | | | | 0.2 mM |  |  |
|  | Casitone | | | | | | | 0.2 % |  |  |
|  | L-cysteine | | | | | | | 4.1 mM |  |  |
|  | Sugar | | | | | | | 0.5 % |  |  |
|  | Mineral solution | | | | | | | 0.61 ml / L |  |  |
|  | Vitamin solution | | | | | | | 0.61 ml / L |  |  |
|  | | | |  | | |  |  |  |  |
| **Mineral solution** | | **g/L** |  | **Vitamin solution** | | | | | | **mg/L** |
| NaCl | | 18 |  | Biotin | | | | | | 16.4 |
| MgCl_2_ | | 0.4 |  | Cobalamine (Vit B12) | | | | | | 16.4 |
| CaCl_2_ | | 0.4 |  | p-ou-4-aminobenzoic acid | | | | | | 49.1 |
| MnCl_2_.4H_2_O | | 0.2 |  | Folic acid | | | | | | 82 |
| CoCl_2_.6H_2_O | | 0.02 |  | pyridoxamine dihydrochloride | | | | | | 246 |
|  | |  |  | Thiamine | | | | | | 82 |
|  | |  |  | Riboflavine | | | | | | 82 |
