# Supplementary material for: A Novel Tightly Regulated Gene Expression System for the Human Intestinal Symbiont Bacteroides thetaiotaomicron
Source: Front Microbiol. 2016 Jul 13;7:1080. doi: 10.3389/fmicb.2016.01080 (PMC4942465; doi:10.3389/fmicb.2016.01080)
Supplement: Supplementary file 2 [file Table2.DOCX]

Supplementary Material

**A Novel Tightly Regulated Gene Expression System for the Human Intestinal Symbiont** *Bacteroides* *thetaiotaomicron*

**Nikki Horn, Ana Lucia Carvalho, Karin Overweg, Udo Wegmann, Simon R. Carding, Régis Stentz***

*** Correspondence:** Corresponding Author: regis.stentz@ifr.ac.uk

| **Table S2.** Oligonucleotide primers used in this study. | |
| --- | --- |
| **Name** | **Sequence^a^** |
| f-noPpepI | ATATAT*GCATGCCCATGGCTCGAGAAAAGCGCT*CCCATATAAAAGAAAAGACACCATGC |
| r-ppepI/NotI | ATGACCTG*GCGGCCGC* |
| f-3784_3786 | ACCGCACCTCCAATAAATAACAGG |
| r-3784_3786_sp | TGAC*GCATGC*AATGTTTTTTCATGGCATAGAATCC |
| f-RBS_low_-pepI | **ATTATAAGGAGGCACTCACCAT**GCAAATCACAGAAAAATATCTTCC |
| r-3784_RBS_low_ | **ATGGTGAGTGCCTCCTTATAAT**ACCGCACCTCCAATAAATAACAGG |
| f-RBS_med__MCS | AGTAC*CCATGG*TGTCTTTTCTTTTATATGG |
| f-RBS_low__MCS | AGTAC*CCATGG*TGAGTGCCTCCTTATAATACCGCACCTCCAATAAATAACAGG |
| ccR_amont2 | CATG*CATATG*AGCTCCATGCTATAGCTACC |
| ccR_aval2 | CATGGGATCCGCCAGCCGTTATGCGGCAGC |
| Tev-His6_linker_5' | CATGGAGGCCTGAGAACCTGTACTTCCAATCCGCTGGACACCACCATCATCATCATTAACCC |
| Tev-His6_linker_3' | GGGTTAATGATGATGATGGTGGTGTCCAGCGGATTGGAAGTACAGGTTCTCAGGCCTC |
| Lactamase_F | CGCTCATTCATCCTGCTTC |
| Lactamase_EcoRI_R | ATAT*GAATTC*TTATTGATGCGTCACA |
| B-Lact_nostop_R | TTGATGCGTCACATATTCG |
| ^a^ The underlined sequence regions match their template. The sequence in italics shows restriction endonuclease recognition sequences. The sequence parts in bold designate the overlap in the splicing by recombinant PCR | |
