# Supplementary material for: A Novel Tightly Regulated Gene Expression System for the Human Intestinal Symbiont Bacteroides thetaiotaomicron
Source: Front Microbiol. 2016 Jul 13;7:1080. doi: 10.3389/fmicb.2016.01080 (PMC4942465; doi:10.3389/fmicb.2016.01080)
Supplement: Supplementary file 3 [file Presentation1.PDF]

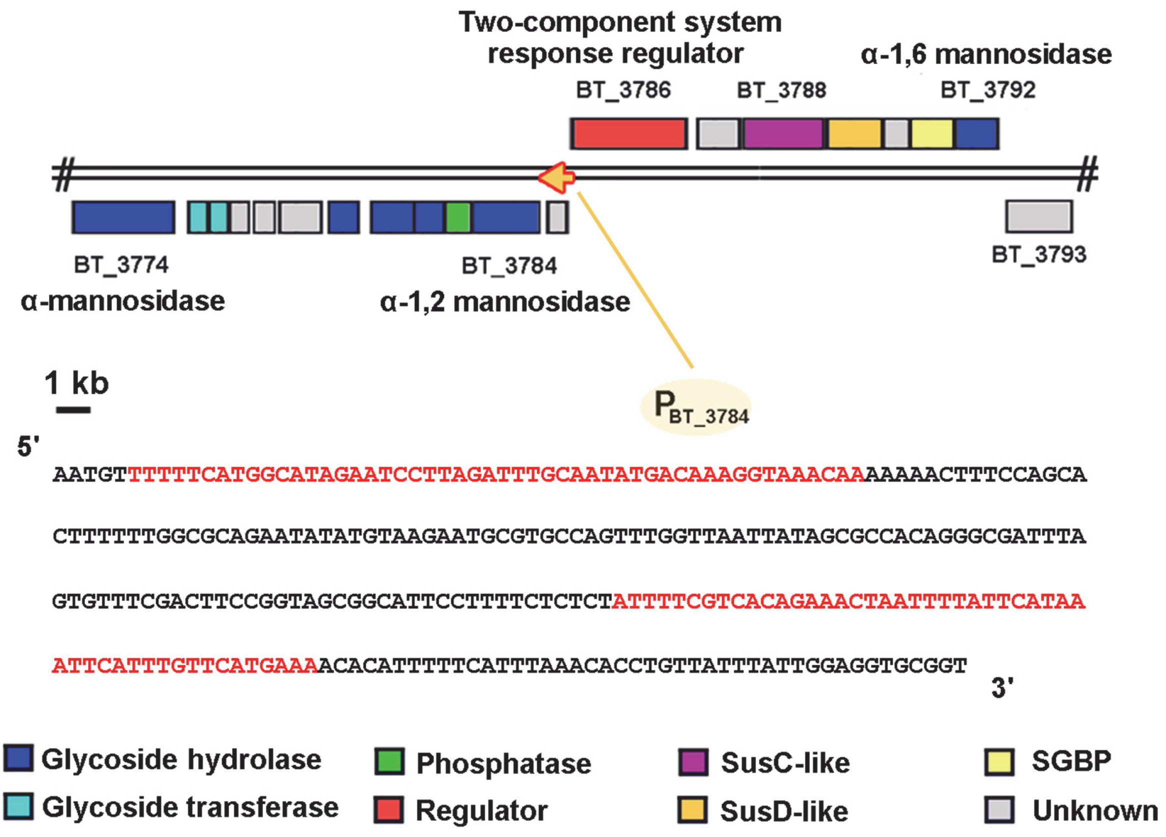

Presentation 1. Genetic map of the *B. thetaiotaomicron* Group 2 PUL BT3774-92. Genes with known or predicted functions (according to Cuskin et al., 2015 and Xu et al., 2003) are colour-coded and genes with unknown function are represented in grey. SGBP; surface glycan binding protein. The sequence and orientation of the P3784 promoter region are indicated. Predictions of promoter sequences with scores between 0.9 and 1 are shown in red and were obtained using the Neural Network Promoter Prediction analysis software (http://www.fruitfly.org/seq_tools/promoter.html).
